# Works and Memoirs on General Psychology

**Published:** 1849-01-01

**Authors:** 


### A BRIEF NOTICE OF THE MOST IMPORTANT WORKS AND MEMOIRS ON PSYCHOLOGY PUBLISHED ABROAD DURING THE YEARS 1846 AND 1847.

(*Condensed, for the most part, from* Amelung's Reports.)

Our object in the following pages is to give our readers a succinct report of some of the most practically useful works on psychology that have appeared on the Continent during the years 1845 and 1846; and to afford those who have not the time or opportunity of examining the great mass of French and German psychological literature, some idea of its general character. We have purposely omitted all reference to works already noticed in our Journal, and to such as treat of the condition of the insane in this country, since the purpose we have in view, as we have already implied, is, if possible, rather to guide the English reader towards new sources of knowledge, than to refer to those with which he is already familiar. By excluding all notices of the French, and, more especially, German memoirs that treat of the psychological literature of Great Britain, our task has been considerably lightened, for the management of the insane adopted in the best of our public and private asylums continues to serve as a model for the institutions of other countries, and constitutes a very considerable portion of the subjects treated of in the numerous works on psychology with which the Continental press is continually teeming.

The first work to which we would refer is "A Manual of the Pathology and Therapeutics of Mental Diseases, by Dr. Schnitzer," * in which the author gives a general summary of the opinions and observations of a very large number of ancient and modern writers on psychology, and as such it constitutes a very useful compendium for the student or practical physician, who would acquaint himself with the works that have from time to time appeared in relation to mental diseases. The author does not, however, furnish us with any new facts or original views; and the value of his work is simply, as we have already remarked, that of being a practical compendium of the labours of others.

The next work on our list,† by Dr. L. F. Calmeil, physician to the

* Handbuch der Pathologie und Therapie der Geisteskrankheiten. Für praktische Aerzte und Studirende bearbeitet von Dr. A. Schnitzer. Leipzig.

† De la folie, considérée sous le point de vue pathologique, philosophique, historique et judiciaire, depuis la renaissance des sciences en Europe jusqu'au dix-neuvième siècle; descriptions des grandes épidémies de delire simple ou compliqué qui ont atteint les populations d'autrefois, et regné dans les monastères. Exposé

Charenton, is a most interesting historical and judicial report of the facts recorded in various public archives, and in authentic private documents, from the fifteenth to the close of the eighteenth century, in relation to dæmonomania, lycanthropia, chorea epidemica, monomania religiosa, &c. On reading these curious records of the benighted condition of former ages, we scarcely know whether we should be more astonished at the superstition and fanaticism which proved such constant and fruitful sources of a disordered imagination (closely akin to actual insanity, and based probably on a tendency to convulsive and epileptic attacks, or on chronic affections of the whole nervous system); or at the ignorance and bigotry of physicians and judges, who suffered those afflicted with such diseases to expiate their miseries at the gibbet or the stake. In looking back with honest pride at the advance we have made over former times in the treatment of mental diseases, we must not, however, forget that it behoves us to be moderate in our rejoicings, as long as the history of psychology continues to record such instances of fanaticism as those which occurred some years since in Switzerland, those of the *incubus* disease in France, the epidemic dæmonomania which prevailed a short time since in Sweden and Russia —besides many others witnessed even occasionally in this country, no less than elsewhere.

M. Pidoux, in a review of M. Brierre de Boismont's work, "On the Acute Delirium of the Insane,"* expresses the opinion that delirium may be associated (as a nervous cerebral affection) with chronic insanity, no less than with other diseases; as, for instance, meningitis, typhus, &c. M. Pidoux, in the first place, regards delirium as a peculiar affection of the brain, more especially of the organs by which thought is regulated, and does not consider it identical with, or dependent upon, inflammation, loss of blood, or any other known cerebral diseases. He, however, looks upon delirium more as a symptom than an actual disease.

In the "Annales med. psychol.," tom. vi., Nov. 1845,† we find an useful report, by Morel, of the psychological journal literature of Germany, from the older journals of Moritz, Reil, Kaissler, and Hoffbauer, to the latest psychological periodicals.

des condemnations auxquelles la folie meconnue a souvent donné lieu. Par L. F. Calmeil, Docteur en Médecine de la Faculté de Paris, Médecin de la Maison de Charenton, &c. T. 2. Paris, 1845.

* De la folie, considérée comme maladie à l'occasion de l'ouvrage suivant: "Du delire aigu observé dans les établissements d'aliénés, par M. Brièrre de Boismont, &c." Par Pidoux. Journal de Méd., par Trousseau. 1845. Dec.

† La Pathologie mentale en Belgique, en Hollande, et en Allemagne. Des Journaux de Psychiatrie en Allemagne. (An. med. psych., t. vi. Nov. 1845.)

In a second memoir (Annales med. psychol., tom. vi., Jan. 1846,*) Morel enters into a full description of the principal asylums of Italy; as for instance, those at Venice, Milan, Genoa, Bologna, Ferrara, Florence, Rome, Naples, and Palermo. This memoir, which evinces great acuteness of observation, and a sound critical spirit, will be read with much interest. Although we learn from M. Morel's report, that the asylums for the insane in Italy do not enjoy the same degree of careful superintendence manifested in the other charitable institutions of that country, a general tendency towards improvement is yet everywhere perceptible. We gather no new facts of practical importance from the results of M. Morel's observation of the mode of treatment adopted by the psychologists of Italy. In the insane asylum for women at Venice, the author found that, of the 1073 patients admitted between the years 1837 and 1843, 215 suffered from pellagra. The institutions at Ferrara and Bologna are very defective. That at Florence is a fine roomy building. Amongst the patients, there were likewise patients with pellagra from districts in the Apennines, where the people do not live on maize.

At the fifth meeting of the Congress of Science, in Italy, four subjects were proposed by the Medical Section:—

1. A classification of mental diseases: to be based on the facts deduced from clinical observations, and pathological anatomy.

2. Whether, and how far, phrenology is able to further our knowledge of mental diseases?

3. Whether, and how far, pathologico-anatomical changes can be regarded as the cause or effect of mental diseases?

4. What symptoms manifest themselves in different mental diseases, which indicate a prophylactic, therapeutic, moral or physical mode of treatment, and of what value are such symptoms?

In a work intended as a reply to these questions,† Professor Carlo Speranza, former President of the Medical Section, enters into an extended disquisition, the purport of which is to prove, that phrenology has not been of use in furthering the study of mental diseases. The third question is left as undecided as it was before; and the fourth is

* La Pathologie mentale en Italie: lettre à Mons. le Docteur Ferrus. Coup-d'œil sur les principaux établissements d'aliénés. (Ann. Med. Psychol., t. vii. Jan. 1846.)

† Riporta al Secondo quesito proposto dalla sezione medica del V. Congresso Scientifico Italiano de Cav. Prof. Carlo Speranza, membro corrispondente di illustri Academie et Società scientifiche, già Presidente della suddetta medica sezione. (Giornale per servire ai Progressi della Pathologia et della Therapeutica, t. x. lev. 2.)

treated in accordance with the present condition of psychological science, but without any new views being advanced.

Leopold Turk's work* demands some notice, from the singular views of the writer, who regards delirium as a purely nervous phenomenon, occasioned by over-excitement of the cutaneous surface with electricity. With the view of curing this affection, Turk recommends the prolonged use of lukewarm baths, continued for days together; having at the same time recourse to venesection, emetics, purgatives, &c. By means of the employment of such baths, the author professes to have cured four-fifths of all his patients.

Dr. C. W. Ideler,† directing physician to the insane department of the Charité Hospital at Berlin, attempts, with perhaps too plausible a form of argument, and with too great scepticism, in reference to the special investigations of anatomy, chemistry, and microscopy, to prove that the salvation of psychology, generally and specially, is to be sought in an anthropology; not such as we are accustomed to regard it, but in the recognition of the influences exercised by all branches of human pursuits combined, as theology, jurisprudence, medicine, and philosophy. This, indeed, is a noble object for the attainment of the psychologist.

Engelken's work‡ is distinguished for the unprejudiced, rational views it advances. In opposition to the majority of psychologists, the author regards the prognosis of mental disease as less unfavourable than that of many other chronic affections. We also differ from him in this, and in the favourable character which he attaches to the prognosis of puerperal insanity, which is often extremely difficult of cure, and not unfrequently wholly incurable. As the superintendent of a private institution, the author naturally gives the preference to such establishments over public institutions. He is averse to all coercive measures, and thinks that artifice is preferable to force in inducing the patient to enter an asylum. His remarks on the duties of the physician who undertakes the charge of the insane, are a mere recapitulation of the views now so generally recognised on this subject. We find, however, that his mode of treatment presents several novel features. Thus, for instance, he adopts a modified form of water-cure; enveloping the patient in wet linen sheets, and applying cold moist cataplasms to the abdomen, in different cases of convulsions; and,

* Mémoire sur la Nature de la Folie, et sur le Traitement à lui opposer. Par Leopold Turk. Paris. 1845.

† Ueber das Verhältniss der Seelenheilkunde zu ihren Hülfswissenschaften, von Dr. C. W. Ideler, Prof. der Med. und dirigirender Arzt der Irrenabtheilung an der Charité zu Berlin. (Allgemeine Zeitschrift für Psychiatrie, iii. B. 3 H. s. 394.)

‡ Beiträge zur Seelenheilkunde, von Dr. Fried. Engelken, Director der Privat. Heilanstalk zu Oberneuland bei Bremen. Bremen. 1846.

lastly, employs electricity, from which he states that he has derived much aid in cases of imbecility, erethism, nervous derangement, and hysteria. Dr. Engelken is a great advocate for the use of opium, not only in melancholia hysterica, but in all instances of extreme irritability of the nervous system where there is erethism, and even in many forms of mania. According to his views, this remedy exercises an invigorating and exhilarating action on the brain and mind. In Dr. Engelken's observations on the psychical treatment of the insane, although we meet with no striking novelties, we are nevertheless forcibly impressed with the proofs of experience and profound thought manifested by the author.

Dr. Michéa's work on Hallucinations and Illusions, to which the Royal Academy of Medecine at Paris* awarded its prize in 1846, is so comprehensive, that it may be said to embrace almost all forms of insanity. We do not agree with the author's views of delirium, which he divides into three kinds, viz. delirium of the senses, of the ideas, and of the passions ; the difference between delirium and insanity depending mainly, according to his definition, on the longer or shorter time during which either continues—delirium being converted into insanity when the mind loses the power of recognising and controlling its wanderings. In like manner we think that Dr. Michéa, like many other French physicians, gives too wide an extension to the idea of hallucinations, under which it would appear that he comprehends almost every form of ideas and conceptions. Thus, French writers discover this symptom in two-thirds, or in half the whole number of the insane; and Esquirol, even in as many as eighty in the hundred. According to M. Michéa himself, the number is five-eighths. The greater number of these cases occur in monomania and general mania—fewer in dementia, and fewer still in imbecility. We cannot take leave of M. Michéa's work without expressing our opinion of its being the most complete treatise that has as yet appeared on this subject. It bears ample testimony to the extended reading and the industry of the author ; and the examples advanced in illustration of his views are so numerous as to be almost wearisome.

Next in order of succession comes Effenberger's memoir,† "On the Present Condition of Psychology in England," to which, as our object is here simply to show what has been done abroad during the last two

* Du delire des Sensations. Par C. F. Michéa, Dr. en Médecine de la Faculté de Paris. Ouvrage couronnée par l'Academie Royale de Médecine. Paris. 1846.

† Der gegenwärtige Zustand des Irrenwesens in England. Geschildert nach Forbes Winslow, von Dr. Joseph Effenberger, K. K. Districts-Physiker zu Bruck an der Leytha in Niederösterreich. (Med. Jahrb. des östr. Staats. Mai.)

years, we simply refer, for the purpose of observing, that Dr. Effenberger's work is based on a treatise by the editor of this Journal, entitled, "An Act for the Regulation of the Cure and Treatment of Lunatics. With Explanatory Notes and Comments."

The literature of Germany, notwithstanding the political disturbances by which the country is shaken, affords evidence of the good results yielded by the free discussion of the state and condition of the insane at the meetings of the German scientific associations, and the philanthropic opinions, based on scientific principles, which have been advanced in Jessen's paper,* read before the Association at Kiel, offer a pleasing contrast to the views entertained in the generation immediately preceding our own.

In turning from *general* to *special* psychology, we must in the first place notice the interesting work of Hohnbaum,† "On Psychical Health and Disease, in their various Transitions." The author follows the almost imperceptible transitions of psychical health, through all the varieties of eccentricities and peculiarities (so often manifested in the every-day life of weak-minded persons and geniuses, in tall and in short men, &c.), to folly or to mania. The author, whose own extensive experience and reading furnish him with numerous illustrations, seeks the cause of mental derangement in some abnormal relations of the body, and is further of opinion that such abnormal somatic relations may be, in the first place, occasioned by a misdirection of the mind, by evil habits, defective education, or the indulgence of the passions.

Erlenmeyer,‡ in a recent memoir, enters into the consideration of the question, whether the blood of the insane presents any peculiar abnormal properties; and whether certain diseases tend towards insanity, or afford immunity against it. The conclusions arrived at are only of a negative character. In the next place, the author considers the different abnormal blood-crases in the insane—viz., the fibrinous, the venous, the serous, and the scorbutic. From the result of 304 dissections, made by the author, at the insane asylum at Prague, it would

* Ueber die in Beziehung auf Geistes-und Gemüthskranke herrschenden Vorurtheile. (Vorgetragen in der allgemeinen Versammlung der deutschen Naturforscher und Aerzte in Kiel am Montage den 21 Sept. 1846. Von Dr. P. Jessen, (Allgem. Zeitschrift. für Psychiatrie, iv. Bd. 1. H.)

† Psychische Gesundheit und Irrensein in ihren Ubergängen. Ein Versuch zur näheren Ergründung zweifelhafter Seelenzustände, fur Criminalisten und Gerichtsärzte. (Von Dr. C. Hohnbaum. Berlin, 1845.)

‡ Ueber das Blut der Irren. Von Dr. Erlenmeyer, erstem Assistenten an der Irrenheilanstalt zu Siegburg. (Archiv. für Physiologische Medicin, v. Jahrg. s. 436, 684.)

appear that the fibrinous (tuberculous) crasis was the most frequent in the insane (134 in 304). Different writers, however, advance such different views regarding this question, that we seem scarcely able, in the present day, to arrive at any very definite conclusion.

Fr. Nasse* attempts to define the local and immediate causes of the various forms of mental derangement. According to his definitions, high delirium is a morbidly excited sensibility of the cerebral organs, which serve to give expression to the psychical emotions—low delirium is a morbid obtuseness of the susceptibility of the brain—chronic mania, an abnormal irritability of the brain, generally the result of a morbid character of the blood—madness (fixed delusion), in a diminished susceptibility of the brain—folly (using the word in its original sense of fatuity), is the result of a diminished power of mental activity and vigour—and imbecility, psychical feebleness in its more highly developed form. Nasse's work has been answered by a memoir, bearing the same title,† from the pen of D. Focke, who endeavours, and we think satisfactorily, to refute the views set forth by Nasse.

Bergmann, in a treatise on the derangements of mind arising from disappointed affection,‡ makes the observation, that in persons (more especially women) this species of mental alienation has generally been preceded by a derangement in the somatic relations of the body, most frequently by a morbid condition of the tissue of the lungs. He further observes, that the organs of respiration are the first to suffer by the psychical disturbance, and that death from pulmonary consumption is a very frequent termination of such affections. The author, who is distinguished for his profound anatomical researches, is of opinion, that erotomania proceeds especially from the cerebellum, and physical love from the organs of sense. The anatomical results appended at the termination of the work, give evidence of the destructive influence occasioned in the organism by moral agencies.

Among the works that have appeared on the Continent, within the period of time included in our considerations, on the physical treatment of the insane, we will in the first place refer to Dr. Weber's memoir, on the effects of certain medicines on the disposition and

* Die Entscheidung über die Unheilbarkeit eines Irreseins. Von Fr. Nasse. (Allg. Zeitsch. für Psychiatrie, iii. 4 H. s. 589.)

† Die Entscheidung über die Unkeilbarkeit eines Irrenseins. Von D. Focke, zweitem Arzte der Irrenheilanstalt zu Siegburg. (Allgem. Zeitschr. für Psychiatrie, iv. Bd. 2, H.)

‡ Bermerkungen über die durch getäuschte Liebe erzeugte Seelenstörung. Von Dr. G. H. Bergmann, Kgl Medicinaerath und Director der Irrenanstalt.

on the sensorium,* in which he treats especially of the good effects produced by agaricus muscarius, anacardium orientale, assafœtida, and belladonna.

Osiander† gives several cases of cure of nymphomania by means of tartar-emetic ointment, and the internal employment of camphor. The latter remedy, given in clysters, was also found highly serviceable in puerperal mania.

Gizzi, as we learn from the Gazette Médicale de Paris, has employed helleborus niger with success in mania and melancholia. The remedy was gradually increased from small doses, and in all cases, although slow, the effect terminated in a perfect cure.

Professor Rech,‡ while he endeavours to establish the efficacy of cold-water affusions, applied to the head, in cases of insanity, admits, however, that these means ought to be practised with much caution, and that they are counter-indicated in acute mania, hallucinations, and other active affections of the brain. The author is opposed to Leured's theory, which ascribes a moral influence to these means; and inclines to the belief that they exercise an undoubted dynamic and material effect.

We find that Aubanel, physician to the Insane Asylum at Marseilles, has invented a bed for uncleanly insane patients, which practical experience has shown to be admirably adapted to the purposes for which it is designed. It consists of a bedstead with high sides, forming a species of trough, having a stout oak bottom, supplied with a grating in the middle, somewhat hollowed out. Below the bottom there is a zinc vessel, which pulls out like a drawer, and may be removed as often as necessary, without in any way disturbing the patient. The bedding consists of finely chopped straw, covered with a sheet. This bedstead is also furnished with a concave cover, having a grating and air-holes, which may be fastened on at will, by means of hinges attached to the sides of the bed.

The general statistical reports of the insane, in Germany and France, afford ample evidence of the improvements, both in public and private asylums, by which the last few years have been characterized in those countries. Among the numerous works, memoirs, and reports that

* Bermerkungen über die Wirkungen einiger Arzneimittel auf das Gemüth und Sensorium. Von Dr. Weber, in Hannover. (Zeitschrift für die gesammte Medicin. herausg. von F. W. Oppenheim.)

† Glossen und Marginalien. (Zeitsch. für die gesam. Med.) Von Oppenheim.

‡ De la douche et des affusions d'eau froide sur la tête dans le traitement des aliénations mentales. Par le Professeur H. Rech, Médecin en chef de l'asile public des aliénés à Montpellier.

have been published in such rapid succession on this subject, we may instance that of M. Cazenave,* which gives a general and comprehensive view of the progress made in France, in the treatment of the insane, since the publication of the well-known edict of the 30th of June, 1838.

We gather from an epistolary communication of Dr. Flemming, that the asylum for the insane at Copenhagen† is still in a very imperfect condition, and that the care and proper treatment of the insane are much neglected throughout the whole of the Danish dominions.

Among the mass of statistical reports before us, referring simply to some circumscribed locality, we would call attention to the admirable memoir of Dr. G. H. Bergmann,‡ which besides the customary statistical tables of the condition of the asylum for the insane at Hildesheim, in the year 1845, enters into the general psychological consideration of the nature of mental diseases generally, of the pathology of the cerebral organs, with observations on epilepsy and phrenology, and on the influence of the weather on the insane.

Dr. Bergmann found that where there had existed a tendency to suicide, a decidedly abnormal condition of the brain was made manifest on dissection. In like manner, there were always morbid changes in incurable cases of imbecility.

The author's definitions of the conjectured seat of the various species of mental disease, although perhaps occasionally somewhat too fanciful for our English mode of reasoning, are nevertheless extremely interesting, and well worthy attentive consideration.

From a notice in the Annales Méd. Psych.§ of the statistics of insanity in Canada, we learn the curious fact, that in Lower Canada the number of idiots exceeds that of the insane threefold, whilst in Upper Canada the numbers of the insane are considerably higher than those of idiots. Can this preponderance of idiotcy, among the Lower Canadians, arise from their Celt-like practice of subdividing the land, and the consequent stationary habits of the people, and their constant intermarriages amongst their own kindred?

* Rapport sur l'asile public des aliénés de Basse-Pyrenées, par M. le Dr. Cazenave, Médecin Directeur. Pau, 1845.

† Zeitschrift für Psychiatrie, iii. Bd. 1, H. s. 181.

‡ Charakteristische Uebersicht der im Jahre, 1845, in die Heil-und Pflegeanstalt zu Hildesheim aufgenommenen Seelengestörten, nebst soustigen die Psychopathologie betreffenden Erörterungen. Von Medicinalrath Dr. G. H. Bergmann. (Hann. Ann. f. d. ges. Heilkunde, vi. J. vi. H.)

§ De la folie en Canada (Annales en Canada, Ann. Méd. Psych. Juillet, 1846.)

It appears from the report of Lillybridge,* that in a population of 20,000 Cherokees, there was not one affected by insanity.

In a statistical report of insanity in Norway, by Dr. Holst,† we find that in 1835, the whole number of the insane was 3576, of which no fewer than 1698 were idiots. In the year 1845, the census showed that the whole number had increased 1667, or about 64·8 per cent., so that while, at the first-named census, there was 1 insane person to 550·7 inhabitants, at the present time there is 1 to 334·1; an increase that is mainly ascribed to the increased consumption of brandy.

---

### Selections.

* Du peu de fréquence de l'aliénation mentale chez les Indiens Cherokee et les Africains. (Ann. Méd. Psych. Juillet. 1846.)

† Ueber die Anzahl der Geisteskranken, Blinden und Taubstummen in Norwegen, im Jahre, 1835. Von Dr. F. Holst. (Allg. Zeitch. f. Psychiatrie, iv. Bd. 3, H.)

